# Operative techniques to reduce anastomotic recurrence in Crohn’s disease

**DOI:** 10.1007/s10151-022-02569-1

**Published:** 2022-01-27

**Authors:** D. Selvakumar, A. E. Sayers, S. R. Brown, L. Hancock

**Affiliations:** 1 Department of General Surgery, Manchester University NHS Foundation Trust, Manchester, UK; 2 Department of General Surgery, Sheffield Teaching Hospitals NHS Foundation Trust, Sheffield, UK

In this video, we demonstrate two operative techniques used to reduce the risk of anastomotic recurrence in Crohn’s disease.

It has been suggested that the mesentery is a pathological driver of disease recurrence in Crohn’s and performing a radical mesenteric excision has shown promising results in reducing rates of anastomotic recurrence [1, 2]. The first section of the video demonstrates a standardised technique to perform a radical mesenteric excision during an ileocaecal resection for terminal ileal Crohn’s disease.

The Kono-S is an antimesenteric, functional, end-to-end, handsewn anastomosis that has been shown to be a safe and effective technique to reduce the risk of anastomotic recurrence [3, 4]. In the second part of the video, we demonstrate a step-by-step approach to performing this as part of an ileocaecal resection, with primary anastomosis, for terminal ileal Crohn’s disease.

## Supplementary Information

Below is the link to the electronic supplementary material.

Supplementary file1 (MP4 1004690 KB)
